# A large osteoderm-bearing rib from the Upper Triassic Kössen Formation (Norian/Rhaetian) of eastern Switzerland

**DOI:** 10.1186/s13358-022-00244-4

**Published:** 2022-02-23

**Authors:** Torsten M. Scheyer, Urs Oberli, Nicole Klein, Heinz Furrer

**Affiliations:** 1 Universität Zürich, Paläontologisches Institut und Museum, Karl Schmid-Strasse 4, CH-8006 Zürich, Switzerland; 2 Waldgutstrasse 21, CH-9010 St.Gallen, Switzerland

**Keywords:** Saurosphargidae, Sauropterygia, Tethys Ocean, Palaeobiogeography, Late Triassic, Osteoderm, Osteology

## Abstract

An important component of the Alpine vertebrate record of Late Triassic age derives from the Kössen Formation, which crops out extensively in the eastern Alps. Here, we present an isolated and only partially preserved large rib, which carries an osteoderm on a low uncinate process. Osteological comparison indicates that the specimen likely belongs to a small clade of marine reptiles, Saurosphargidae. Members of the clade are restricted to the western (today Europe) and eastern margins of the Tethys (today China) and were so far known only from the Anisian stage of the Middle Triassic. The assignment of the new find to cf. Saurosphargidae, with potential affinities to the genus *Largocephalosaurus* from the Guanling Formation of Yunnan and Guizhou Provinces, China, would extend the occurrence of the clade about 35 million years into the Late Triassic.

## Introduction

The Upper Triassic Kössen Formation is a lithostratigraphic unit at the western end of the Austroalpine, the uppermost tectonic unit of the eastern Alps in Switzerland and adjacent areas in Germany, Austria, and Italy. Especially the lower part of the formation (the basal Alplihorn Member and the overlying Schesaplana Member), is highly fossiliferous. The shallow marine limestones and shales of late Norian to Rhaetian age yielded, besides many invertebrate fossils, a diverse vertebrate fauna (Furrer, 1993; Furrer et al., 1992). The latter are usually represented as isolated or mostly disarticulated skeletal remains (teeth, bones, scales), whereas articulated remains are overall rare (e.g., Grüter, 2006; Neenan & Scheyer, 2014). The fauna comprises cartilaginous and bony fishes (e.g., Bürgin & Furrer, 1992, 1993, 2004; Duffin & Furrer, 1981; Kühn, 1940), and a diverse marine reptile fauna including large to giant ichthyosaurs (Furrer, 1993; Karl et al., 2014; Zapfe, 1976), thalattosaurs (Müller, 2007), and sauropterygians; the latter mainly represented by placodont remains (e.g., Broili, 1920; Grüter, 2006; Kühn, 1940; Neenan & Scheyer, 2014; Schubert-Klempnauer, 1975). Phytosaurs (Furrer, 1993), pterosaurs (Fröbisch & Fröbisch, 2006; Stecher, 2008), and temnospondyl amphibians (HF, pers. obs.) are rare terrestrial faunal elements.

Despite the high diversity of fossil vertebrates, even isolated vertebrate remains from the Kössen Formation are usually diagnostic to the family or genus level. Herein we present a new specimen, identified as a rib and associated osteoderm, representing a shape that so far did not conform to any of the above-mentioned clades. Instead, when expanding the spatiotemporal search into the Middle Triassic, it shows resemblances with other marine and terrestrial taxa such as the non-cyamodontoid placodont *Paraplacodus broilii* Peyer, 1931, the enigmatic *Helveticosaurus zollingeri* Peyer, 1955, the heavily armoured *Eusaurosphargis dalsassoi* Nosotti & Rieppel, 2003 (Nosotti & Rieppel, 2003; Peyer, 1931, 1955; Rieppel, 1989; Scheyer et al., 2017), as well as Saurosphargidae (Li et al., 2011, 2014).

Saurosphargidae, to date a still poorly known family, has been introduced for a clade combining the only European representative, *Saurosphargis voltzi* Huene, 1936 from the Early Anisian of Gogolin, Poland (Huene, 1936), with a single Asian species, *Sinosaurosphargis yunguiensis* Li et al., 2011 from the Middle Triassic (Anisian, Pelsonian) Guanling Formation of Yunnan and Guizhou Provinces, southern China (Li et al., 2011). The paired taxa were initially found to be the sister clade to Thalattosauriformes (Li et al., 2011), but with the description of two more species from the Guanling Formation, *Largocephalosaurus polycarpon* Cheng et al., 2012 and *Largocephalosaurus qianensis* Li et al., 2014, Saurosphargidae has since been recovered as the sister group of Sauropterygia (e.g., Li et al., 2014; Shang et al., 2020). Alternatively, a potential position of saurosphargids within Sauropterygia was proposed (e.g., Cheng et al., 2012; Scheyer et al., 2017). It is noteworthy here that S*aurosphargis voltzi* is based only on a single specimen considered lost since World War II, and because it is only part of an animal’s trunk it has been treated as a nomen dubium in some studies (Nosotti & Rieppel, 2003; Scheyer et al., 2017).

The aim of this short article is to present a brief osteological description and taxonomic identification of specimen PIMUZ A/III 5166. Osteological comparison with other Triassic tetrapod groups is used as basis for exploring palaeobiogeographic implications.

## Geology and stratigraphy

**Fig. 1:**
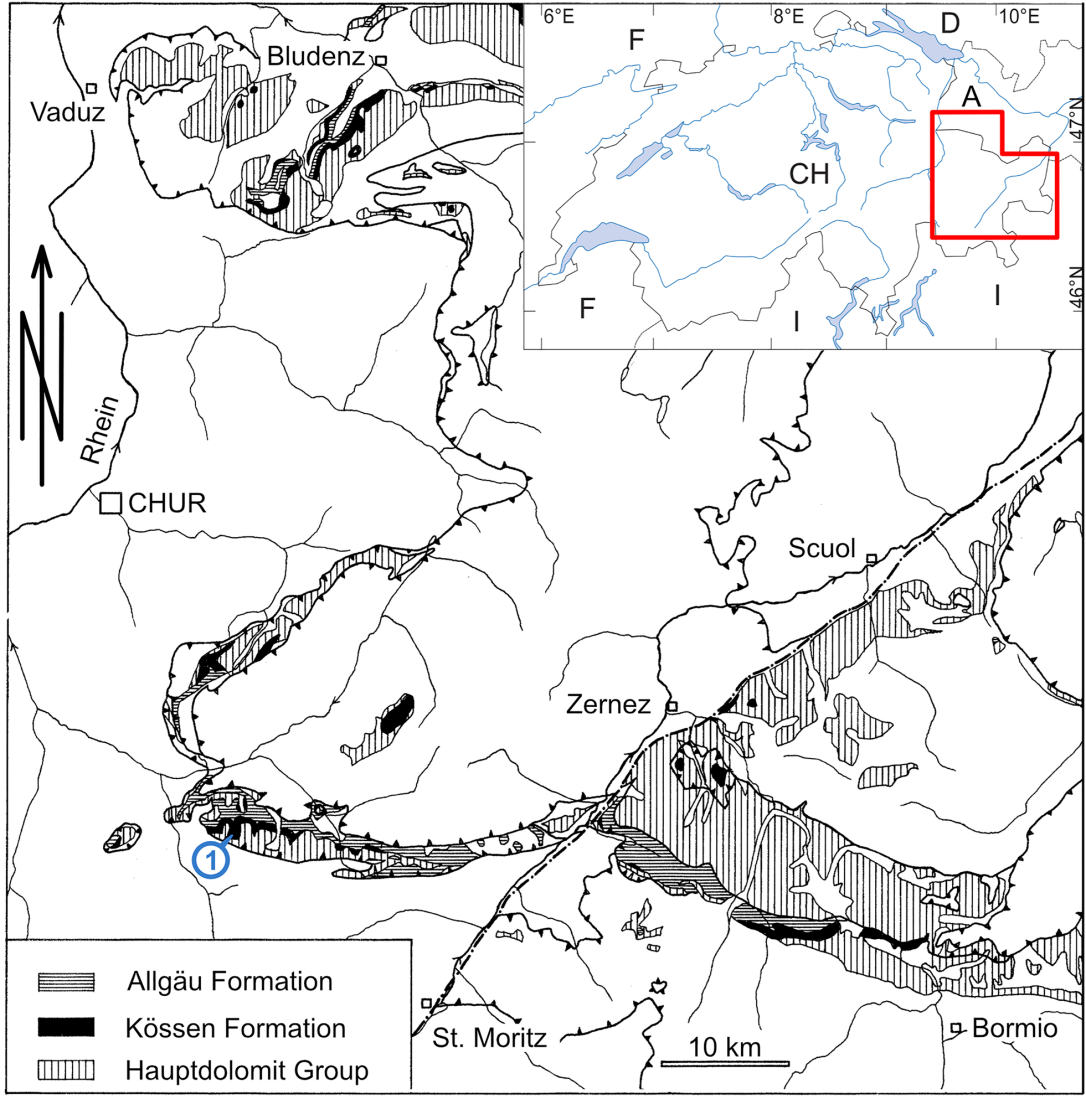
Geographical map and geological context of PIMUZ A/III 5166. The specimen was found in scree (position is indicated by blue circle and a blue 1) of the Upper Triassic Kössen Formation northwest of the mountain Piz Mitgel (lower Austroalpine Ela nappe). A, Austria; CH, Switzerland; D, Germany; F, France; I, Italy

The fossil was found in the scree of a small valley northwest of Piz Mitgel (46°37’15’’ N / 9°38’28’’ E). The grey limestone slab, rich in bivalve shells, is typical for the lower part of the Upper Triassic Kössen Formation, cropping out in the adjacent crest at Point 2684, where a stratigraphic section was studied by one of the authors (HF) in 1973 during geological mapping in the Lower Austroalpine Ela nappe (Fig. 1). The Kössen Formation, well exposed in various Austroalpine nappes of the eastern Alps of southeastern Switzerland (Canton of Grisons) and adjacent western Austria and northern Italy, is subdivided into five members (from bottom to top: Alplihorn, Schesaplana, Ramoz, Zirmenkopf, and Mitgel members; Furrer, 1985, 1993).

Based on the lithology of the matrix block (a bivalve-rich lumachelle), the specimen most probably derives from the Alplihorn or the Schesaplana members, the 50–100 m thick lower part of the Kössen Formation, rich in dark grey shales, marls and limestones. Occasionally, layers of siltstone, oolitic limestone and dolomites are interbedded. The well-bedded marine sediments are strongly bioturbated and rich in bivalves, often concentrated in storm beds (tempestites), typical for a shallow marine basin. The disarticulated bones, teeth and scales of fishes and reptiles are usually found in tempestites (Bürgin & Furrer, 1992, 1993; Duffin & Furrer, 1981; Furrer, 1993). The interbedded coral limestones of the Schesaplana Member with brachiopods, echinoids and crinoids suggest a better connection of the shallow basin to the open sea.

The exact age of the Kössen Formation in the Austroalpine nappes of Switzerland is not well constrained due to the absence of good index fossils such as conodonts, ammonoids, and palynomorphs. Carbonate carbon isotope stratigraphy in the region of Lorüns (Vorarlberg, western Austria; Northern Calcareous Alps) proves a late Rhaetian age for the top of the Kössen Formation (Felber et al., 2015). The Schesaplana Member must have a Rhaetian age as suggested by the Rhaetian foraminifers *Glomospirella friedli* and *Triasina hantkeni* in association with *Rhaetavicula contorta* (Furrer, 1985, 1993). Based on the occurrence of *Rhaetavicula contorta* (Furrer, 1993; McRoberts, 2008, 2010) in particular, a late Norian to early Rhaetian age is likely for the Alplihorn Member.

## Materials and methods

PIMUZ A/III 5166, an isolated and fragmented bone, deep-brown in colour, rests on a slab of greyish limestone rich in bivalves, derived from a scree slope of the Upper Triassic Kössen Formation (latest Norian to Rhaetian) in the Valletta da Mitgel, 1 km northwest of Piz Mitgel, community of Albula/Alvra, Canton of Grisons, eastern Switzerland (46°37’15’’ N / 9°38’28’’ E). The specimen was found by one of the authors (UO) in summer 1998 and subsequently prepared mechanically and by diluted formic acid. The bone is extremely flattened in anteroposterior direction so that its overall thickness ranges only between 1 and 2 mm. The specimen was studied using a Leica MZ16 Stereomicroscope and photographed with a Nikon D2X camera. In addition, the specimen was X-rayed by Dr. Henning Richter and the team at the Diagnostic Imaging Research Unit (DIRU), Clinic for Diagnostic Imaging, Department of Clinical Diagnostics and Services, Vetsuisse Faculty, University of Zurich. Images were taken with a Bucky Diagnost CS/TH X-Ray (Philips) und Profect CS Mammo-Reader (Fujifilm), with 70 kV and 120 mAs.

First-hand osteological comparison was made with the following taxa (in alphabetical order): *Eretmorhipis carrolldongi* Chen et al., 2015: WGSC V26020 (holotype), an almost complete postcranium; *Eusaurosphargis dalsassoi* Nosotti & Rieppel, 2003: MSNM BES SC 390 (holotype), an associated but disarticulated skeleton; PIMUZ A/III 4380, a complete and fully articulated skeleton prepared in ventral view; NMNHL Wijk10-246, an isolated dorsal rib. *Helveticosaurus zollingeri* Peyer, 1955: PIMUZ T 4352 (holotype), a mostly complete and articulated skeleton; PIMUZ T 4353, an associated but disarticulated skeleton. *Largocephalosaurus qianensis* Li et al., 2014: IVPP V15638 (holotype), nearly complete and mostly articulated skeleton prepared in ventral view; GMPKU-P-1532-B, an articulated and mostly complete postcranial skeleton prepared in dorsal view. *Parahupehsuchus longus* Chen et al., 2014a, 2014b, 2014c: WGSC 26,005, a partial but well-articulated skeleton prepared in left lateral view. *Paraplacodus broilii* Peyer, 1931: PIMUZ T 2806 (holotype), a disarticulated but fragmented skeleton; PIMUZ T 4775, a nearly complete and articulated skeleton prepared mostly in dorsal and lateral view; PIMUZ T 4287, an associated and partly articulated partial postcranial skeleton prepared in dorsal view. *Pararcus diepenbroeki* Klein & Scheyer, 2014: TWE 480000454 (holotype), an associated but disarticulated postcranial skeleton. *Placodus inexpectatus* Jiang et al., 2008: GMPKU-P-1054, a complete and articulated skeleton prepared in right lateral view. *Sinosaurosphargis yunguiensis* Li et al., 2011: IVPP V 17040 (holotype), an articulated but only partially preserved skeleton prepared in dorsal view; IVPP V 16076, an associated and largely articulated skeleton prepared in ventral view.

Additional specimens of Middle-to-Late Triassic larger reptiles were studied in person as well, such as sauropterygian, thalattosaur, ichthyosauromorph (ichthyosaurs and hupehsuchians), and archosauromorph specimens from Europe and China (e.g., *Askeptosaurus italicus*: PIMUZ T 4831, 4832, 4846; *Besanosaurus leptorhynchus*: PIMUZ T 1895, 4376, 4847; *Cymbospondylus buchseri*: PIMUZ T 4351; *Dinocephalosaurus orientalis*: IVPP V13767; *Nothosaurus giganteus*: PIMUZ T 4829 [= holotype of *Paranothosaurus amsleri* Peyer, 1939]; *Paratypothorax andressorum*: SMNS 5721*;* Phytosauria indet.: PIMUZ A/III 4368; *Qianosuchus mixtus*: IVPP V13899; *Tanystropheus hydroides*: PIMUZ T 2818; *Ticinosuchus ferox*: PIMUZ T 4779; *Wangosaurus brevirostris*: GMPKU-P-1529; *Yunguisaurus liae*: ZMNH M8738). In addition, isolated osteoderm morphology of phytosaurs, rauisuchians, and aetosaurs was assessed (e.g., Scheyer and Desojo, 2011; Scheyer et al., 2014) and the osteology of the pistosauroid sauropterygian *Bobosaurus forojuliensis* (MFSN 27285) was taken from the literature (Dalla Vecchia, 2006; Fabbri et al., 2014).

Institutional abbreviations: *GMPKU* Geological Museum of Peking University, Beijing, China; *IVPP* Institute for Vertebrate Paleontology and Paleoanthropology, Chinese Academy of Sciences, Beijing, China; *MFSN* Museo Friulano di Storia Naturale, Udine, Italy; *MSNM* Museo di Storia Naturale di Milano, Milan, Italy; *NMNHL* National Museum of Natural History (Naturalis), Leiden, The Netherlands; *PIMUZ* Palaeontological Institute and Museum of the University of Zurich, Zurich, Switzerland; *SMNS* Staatliches Museum für Naturkunde, Stuttgart, Germany; *TWE* De Museumfabriek, formerly Museum TwentseWelle, Enschede, the Netherlands; *WGSC* Wuhan Centre of China Geological Survey, Wuhan, China; *ZMNH* Zhejiang Museum of Natural History, Hangzhou, Zhejiang, China.

## Results

### Systematic palaeontology

cf. Saurosphargidae Li et al., 2011

cf. Saurosphargidae indet.

(Fig. 2).

**Fig. 2:**
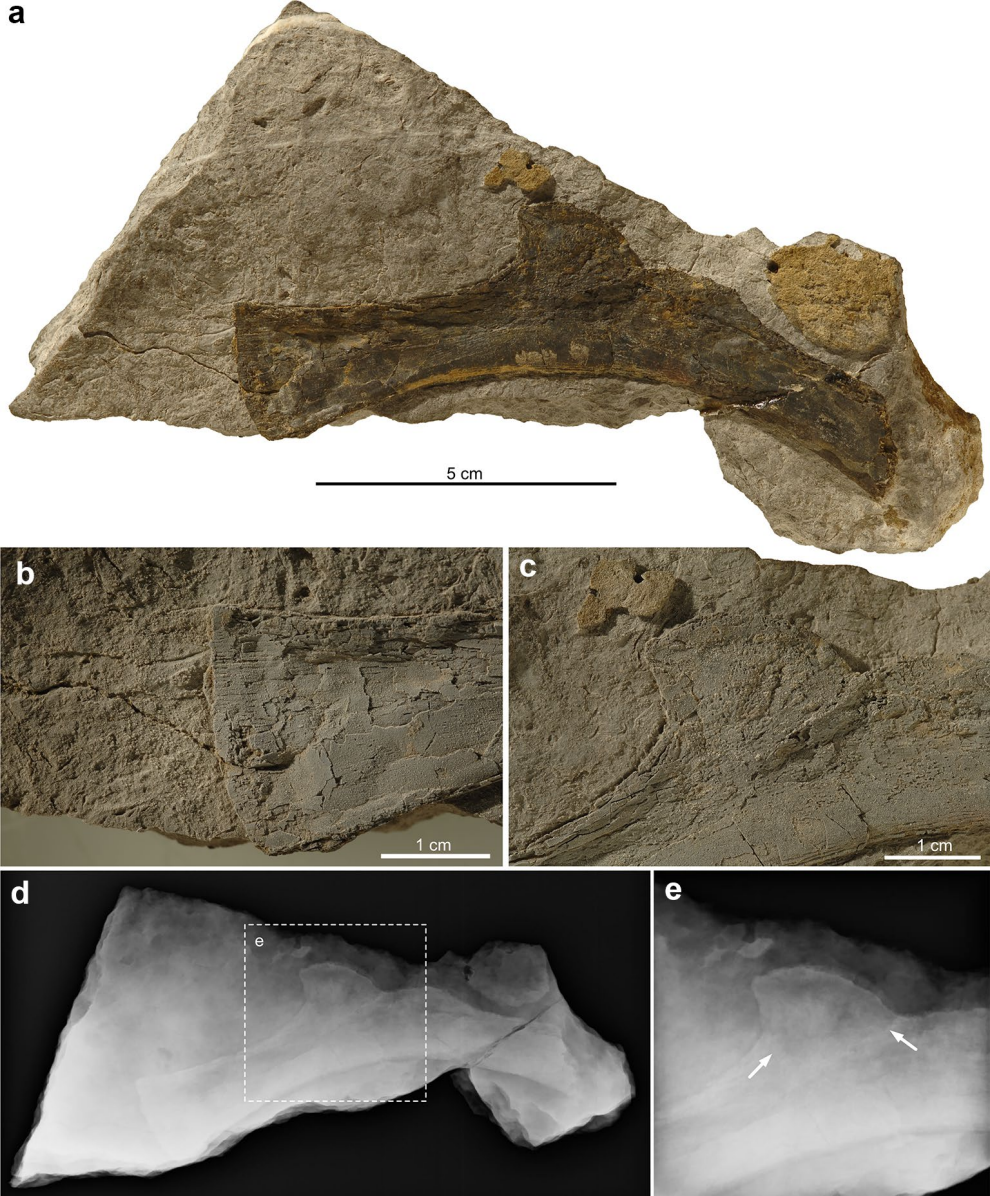
PIMUZ A/III 5166, a large saurosphargid thoracic rib with a low uncinate process and sub-triangular osteoderm. Images in **b** and **c** show specimen coated in ammonium chloride to enhance surface structures. **a** Complete specimen on slab of sediment. **b** Close-up of the slightly expanded holocephalous rib head. Note striation of the bone close to the articular surface. **c** Close-up of the uncinate process and the osteoderm, the latter exhibiting a slightly pitted surface sculpturing oriented differently than that of the rib. **d**, **e** Radiographic image of complete specimen (**d**) and close-up (**e**) of the osteoderm. The boundary of rib and osteoderm is indicated by white arrows

### Osteological description

PIMUZ A/III 5166 preserves two bones on a slab of lumachelle carbonate rock (Fig. 2a–c; Table 1). One end of the larger, incomplete bone is undamaged; ending in a slightly convex articulation surface, the opposite end of the bone shows a clear break. Based on a general curvature with a gentle concave and opposite convex margin, the larger bone fragment conforms to a thoracic rib, which would indicate that the articulation surface constitutes the gently expanding proximal end of a holocephalous thoracic rib. About half-length along its convex margin, the bone also shows a slight trapezoidal expansion, consistent with the expansion of a low flange or uncinate process. Along its length, the bone shows locally some proximodistal ‘striation’ (mostly visible where the bone surface is more damaged), which is likely due to proximodistal extending vascular canals within the bone cortex.

**Table 1:** Measurements of PIMUZ A/III 5166, a thoracic rib and associated osteoderm

Length of main bone fragment (as preserved)	114.85 mm
Height of main bone at unbroken end (expanded articular surface)	23.92 mm
Height of main bone at broken end (distal end)	15.22 mm
Length of trapezoidal expansion (uncinate process)	54.69 mm
Maximum height of main bone at trapezoidal expansion	19.67 mm
Length of additional bone (osteoderm)	19.52 mm
Height of additional bone (osteoderm)	17.44 mm

In addition, there is a sub-triangular bone superimposed on the proximal portion of the expanded area of the longer bone. The additional bone, here interpreted as an osteoderm residing on the low flange of the thoracic rib, shows a slightly raised central area but is otherwise devoid of surficial features due to extreme flatting during fossilisation. The complete separation of both bones was confirmed by the X-rays taken of the specimen (Fig. 2d, e), so a subsurface connection of both elements can be discarded. Furthermore, the sub-triangular bone, i.e. osteoderm, is considered resting in situ on the proximal part of the rather fragile and thin uncinate process, instead of being transported and deposited into this particular position on the rib.

## Discussion

Thalattosaurs and ichthyosaurs completely lack osteoderms and flange-like protrusions of the thoracic ribs (Bindellini et al., 2021; McGowan & Motani, 2003; Sander, 1989), but there are several lineages of extant and extinct vertebrate groups that have thoracic ribs with low flanges to very pronounced uncinate processes. Among those, temnospondyl amphibians (e.g., *Mastodonsaurus giganteus*; Schoch, 1999) and stegocephalians (e.g., *Ichthyostega stensioei*; Ahlberg et al., 2005), *Helveticosaurus zollingeri*, certain sauropterygians (e.g., *Bobosaurus forojuliensis*; Dalla Vecchia, 2006), and bird-lineage dinosaurs (e.g., *Oviraptor philoceratops*; Codd, 2004) lack postcranial osteoderms. Early stem-turtles that lacked rigid shells had broadened dorsal ribs, but these were also not covered by separate osteoderms (Lyson et al., 2014, 2016), whereas rows of usually strongly sculptured osteoderms are incorporated into a broader paravertebral shield supporting the vertebral spine of crocodylians (e.g., Frey, 1988).

Having both holocephalous thoracic ribs with a clearly expanded but not strongly protruding uncinate process and a larger sub-triangular osteoderm associated with such uncinate process reduces the list of potential taxa to armoured Triassic reptiles including certain diapsid taxa such as *Eusaurosphargis dalsassoi*, some sauropterygians and saurosphargids (Klein & Sichelschmidt, 2014; Li et al., 2014; Peyer, 1931). Among those, *Paraplacodus broilii* and *Placodus* spp. lack osteoderms associated with their ribs (Drevermann, 1933; Jiang et al., 2008; Peyer, 1931). In the placodont *Pararcus diepenbroeki*, holocephalous thoracic ribs are known, with them showing only a slight ridge of the rib much less pronounced than, for example, the uncinate processes of *Paraplacodus broilii*. The position of larger osteoderms (some being sub-triangular similar to the one on PIMUZ A/III 5166) on the body cannot be reconstructed with confidence however, because of the disarticulated nature of the only known associated specimen of *Pararcus diepenbroeki* (Klein & Scheyer, 2014). Cyamodontoid placodonts on the other hand carry a turtle-like shell over strongly broadened vertebral transverse processes, whereas their ribs are reduced in size, lack uncinate processes, and are usually confined to the lateral carapace walls (Huene, 1936; Scheyer, 2010). Hupehsuchians have robust rib cages with stout ribs exhibiting flange-like uncinate processes along the mid-shaft region (well visible in specimens of *Eretmorhipis carrolldongi*; Chen et al., 2015; Cheng et al., 2019) and carry an intricate assembly of larger osteoderms, often arranged in interlocking rows, over their vertebral spines (e.g., Carroll & Dong, 1991; Chen et al., 2014a, b, c). There appear also to be small granular osteoderms associated with lateral gastral elements in *Hupehsuchus nanchangensis* as well (Chen et al., 2014a; specimen WGSC 26004). A correlation of larger osteoderms with the ribs, however, is absent in all described hupehsuchians. *Eusaurosphargis dalsassoi*, as indicated by PIMUZ A/III 4380 (Scheyer et al., 2017), shows disparity in thoracic rib shape linked to rib position and the size, angle, and protrusion of their uncinate processes, none of which resemble the shape of PIMUZ A/III 4380. The osteoderms associated with these prominent uncinate processes have a broadened and rounded sculptured base and a tapering apex similar to pylons, whereas the leaf-shaped lateral osteoderms of *Eusaurosphargis dalsassoi* are associated with the gastral ribs, not the dorsal ribs.

**Fig. 3:**
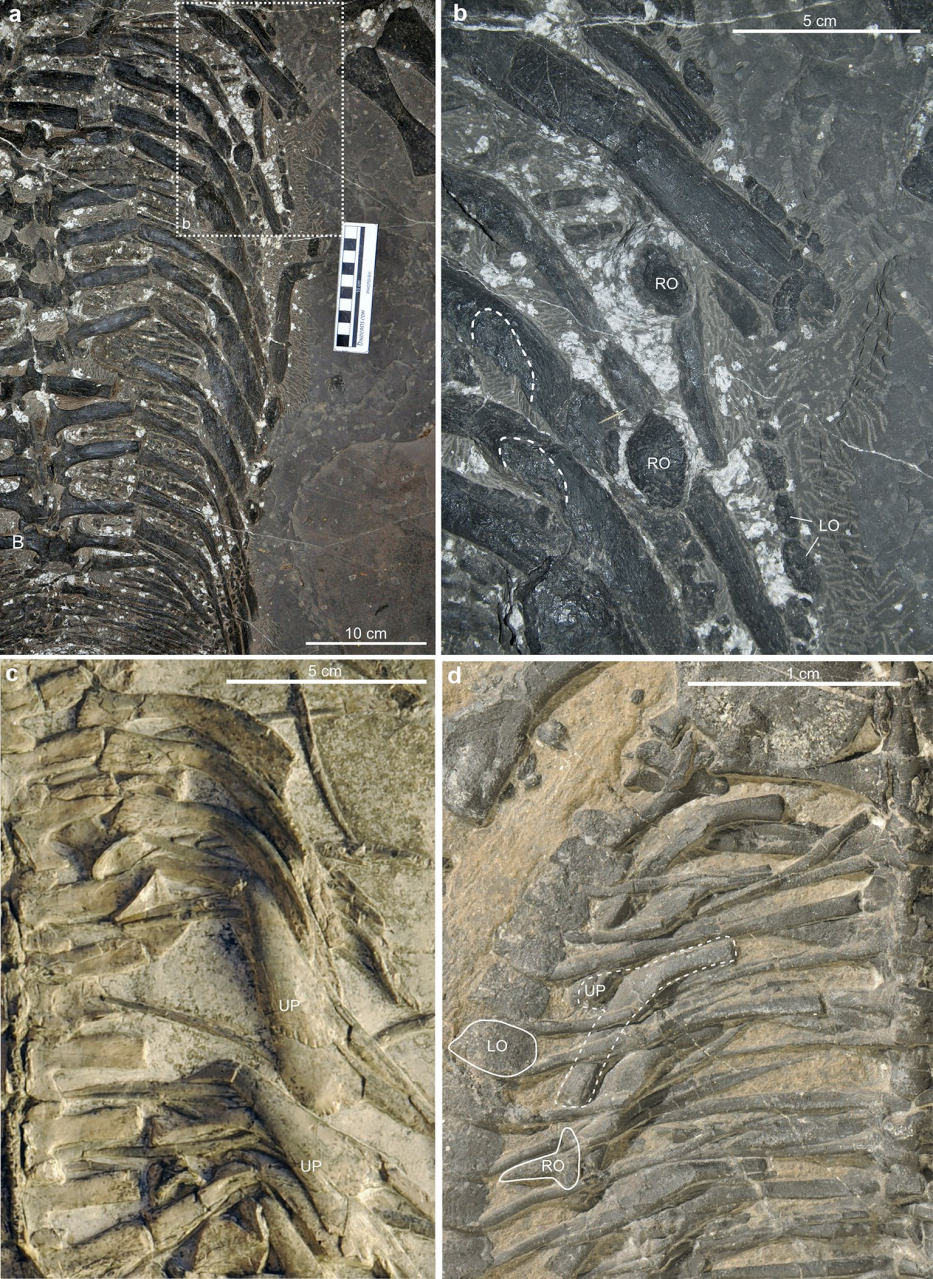
Comparison of the thoracic regions of *Largocephalosaurus qianensis* (GMPKU-P-1532-B; **a** and **b**), *Paraplacodus broilii* (PIMUZ T 4287, **c**), and *Eusaurosphargis dalsassoi* (PIMUZ A/III 4380, **d**). **a** Right half of the rib cage in dorsal view. The rectangle indicates the position of the close-up shown in **b**. **b** Close-up of the anterior ribs and osteoderms. Note rugose and slightly depressed surfaces on the ribs’ uncinate processes (indicated by thin stippled lines), that resemble the position where the osteoderms were positioned in situ. **c** Close-up of the anterior right thoracic ribs shown wide flaring uncinate processes with a smooth bone surface. **d** Rib with uncinate process and pylon-shaped osteoderm. LO, lateral row osteoderm; RO, rib osteoderm; UP, uncinate process

*Saurosphargis voltzi* has closely spaced broad and thick ribs and more hook-like flanges/uncinate processes, and small roundish to elongate shaped osteoderms (Huene, 1936; Klein & Sichelschmidt, 2014; Nosotti & Rieppel, 2003). The proximal articulations of the thoracic ribs are almost straight and lack the expansion (Huene, 1936) seen in PIMUZ A/III 5166. The saurosphargid *Sinosaurosphargis yunguiensis* shows broadened ribs (that lack local uncinate processes) being extensively covered by small osteoderms that form a complete carapace over the trunk and the appendages (Li et al., 2011) and lack larger osteoderms. The saurosphargid *Largocephalosaurus qianensis* has ribs with uncinate processes (Fig. 3a, b), a set of granular-shaped to elongated osteoderms only a few millimetres in dimensions that covered large parts of the trunk and likely the appendages, but may not have formed a complete carapace as in *Sinosaurosphargis yunguiensis*. In addition, *Largocephalosaurus* also has a median row of osteoderms associated with the vertebral spines and two parasagittal rows of larger osteoderms associated with the ribs and likely additional lateral rows (Li et al., 2014). Especially those of the parasagittal rows can have a sub-triangular shape (Li et al., 2014: their Figure 4h). Compared to *Paraplacodus* and *Eusaurosphargis* ribs (Fig. 3c, d), the uncinate processes of *Largocephalosaurus* are less pronounced.

## Conclusions

We describe a new rib and associated osteoderm morphology hitherto unknown from the Alpine Late Triassic. Given the considerations discussed above, and acknowledging the fragmentary nature of the new Alpine specimen, the most plausible interpretation for PIMUZ A/III 5166 is that the thoracic rib and associated osteoderm represent a specimen of cf. Saurosphargidae, similar to *Largocephalosaurus qianensis* from the Anisian of southern China (Li et al., 2011). PIMUZ A/III 5166 is of similar dimensions to the dorsal ribs of the holotype specimen IVPP V 15638 of *L. qianensis.* As such, size comparison with this more completely known animal would indicate that the specimen from the Alps might have derived as well from an animal of about 2.5 m in length.

In this scenario, PIMUZ A/III 5166 would extend the temporal range of the Saurosphargidae about 35 million years from the Anisian (> 242 Ma) Middle Triassic to the Late Triassic (Rhaetian/Norian 205–210 Ma) and, potentially document, if the presence of a *Largocephalus*-like animal could be confirmed by additional specimens, the presence of another eastern Tethyan faunal component in the western Tethys Alpine region; other examples being known in sauropterygian and ichthyosaur genera (e.g., Jiang et al., 2005; Shang, 2006; Wang et al., 2019) and archosauromorphs (Jaquier et al., 2017; Li, 2007; Rieppel et al., 2010; Scheyer et al., 2020).

In contrast to the species-rich and famous Alpine localities of the Middle Triassic, Late Triassic vertebrate remains from the Austroalpine domain (lower and upper Austroalpine nappes, including the Northern Calcareous Alps) at the western end of the Tethys are still considered rarer, so in any event, the osteoderm-bearing rib constitutes a faunal component previously undocumented in the Upper Triassic Kössen Formation of Switzerland.

## Data Availability

All data generated or analysed during this study are included in this published article and the fossil described herein is officially accessioned and available upon request at PIMUZ.
